# Automatic summarization model based on clustering algorithm

**DOI:** 10.1038/s41598-024-66306-4

**Published:** 2024-07-03

**Authors:** Wenzhuo Dai, Qing He

**Affiliations:** College of Big Data and Information Engineering, Guizhou University Guiyang, Room 421, Chongli Building, West Campus of Guizhou University, Jiaxiu South Road, Huaxi District, Guiyang City, 550025 Guizhou Province People’s Republic of China

**Keywords:** EDS, Cluster algorithm, Semantic space, Computer science, Information technology

## Abstract

Extractive document summary is usually seen as a sequence labeling task, which the summary is formulated by sentences from the original document. However, the selected sentences usually are high redundancy in semantic space, so that the composed summary are high semantic redundancy. To alleviate this problem, we propose a model to reduce the semantic redundancy of summary by introducing the cluster algorithm to select difference sentences in semantic space and we improve the base BERT to score sentences. We evaluate our model and perform significance testing using ROUGE on the CNN/DailyMail datasets compare with six baselines, which include two traditional methods and four state-of-art deep learning model. The results validate the effectiveness of our approach, which leverages K-means algorithm to produce more accurate and less repeat sentences in semantic summaries.

## Introduction

Extractive document summary (EDS) is an important task in NLP (Nature Language Processing), which aims to extract sentences from a document to form a summary while keeping information of the original text. In this paper, we focus on the problem which usually generates high semantic redundancy summary.

In recent years, EDS task has achieved great success with the prosperity of neural networks. However, it faces a challenge in high semantic sentences are selected in summary. Previous methods to solve this problem can be divided into two sides: (1) constructing complex architecture network to represent sentences or words separately to reduce the high semantic redundancy problem; (2) using pre-trained model to select sentences with low semantic redundancy.

There are many excellent works based on the first way. For example, Dina et al.^1^ proposed a system with three steps includes answer extraction, semantic clustering and extractive summary. Another approach is proposed by Zhong et al.^2^ They seem the extractive summary task as the text matching task, which using the selected sentences by BERTSUM to formulate candidate set and array the sentences to text match with reference summary. In this paper, we follow the approach as MatchSum and propose using the K-means cluster algorithm to reduce the semantic redundancy in summary.

Cluster algorithms are widely used in semantic spaces, especially in the fields of nature language processing and information retrieval. Common clustering algorithms include: K-means^3^ is a common clustering algorithm that can be used to divide data points in text or semantic space into different clusters. Hierarchical clustering can build a clustering hierarchy based on the similarity between data points, which helps to understand the data organization relationship in the semantic space. DBSCAN is a density-based clustering algorithm suitable for discovering irregularly shaped clusters and discovering implicit semantic relationship in the semantic space. These algorithms can help understand the data structure in the semantic space, discover potential semantic relationships and effectively organize and classify data points in the semantic space.

Traditional method of EDS is extracting sentences individually and modeling the relationship between sentences, then finding top-n sentences as the summary^4^. In recent years, extractive methods for summary have been proven effective in many systems, but the existing extractive models are focusing the score of the sentence and ignoring the sentences may have same semantic, so that the extracted summary have the repeat sentences in semantics. In this paper, we propose an EDS model with cluster algorithm to alleviate the problem of high semantic redundancy in summary. Cluster algorithm have been applied for clustering the vectors into different cluster, so we choose the K-means to filtrate the repeat sentences in semantic space to formulate summary.

Specific to model, we improve BERT architecture to score the sentences of document and select low semantic redundancy sentences with the help of cluster algorithm to formulate summary. The K-means module receive the sentences embedding and the score from the Score-BERT, the K-means module makes the sentences embedding map to high dimension semantic space and choose the difference sentences to formulate summary. We evaluate the proposed EDS model and conduct a significance test using the ROUGE score metrics on the CNN/DailyMail dataset to compare the baseline model.

## Related work

### Extractive document Summary

The EDS task aims to extract sentences from the original document to form a summary. The task first encodes the sentences with the help of an encoder to obtain words vector, then pass a sentence encode module to get a sentence vector to represent a sentence. The sentence vector then passes through a classification layer to determine whether it should be included in the summary. Nallapati et al.^2^; Zhou et al.^5^ chose recurrent neural networks (RNN) for sentence encoding, while Wang et al.^6^ had been using transformer. BERT and other pre-trained language models also perform well in the EDS task.

Although these methods are effective, they mostly did not consider the semantic problem. To alleviate the semantic problem, Mohd et al.^7^ used a distributional semantic to capture and preserve the semantics of text as the fundamental feature for summarizing; Kirmani et al.^8^ utilized bio-semantic models on the domain of bio-medical research; Bhat et al.^9^ used emotion described by text as semantic feature; Kirmani et al.^10^ proposed an email summarizing system by semantic models and deep-learning technologies to summarize emails; Mud et al.^11^ proposed an advanced text document summarizer with cluster algorithm to preserving the underlying semantics of the original text. Although the above methods take into account the semantics of preserving the original text, it does not take into account the problem of redundancy, which may make the sentence semantic redundancy in the extracted summary.

Base on the above extract models and semantic summarizer, we introduce cluster algorithm to classify the sentences by their semantics, which improves model performance and reduce the semantic redundancy in summary.

### Sentence embeddings for EDS

An important step in the task of extracting a document summary is obtaining sentence embedding. The traditional sentence embedding method is based on the weighted and average of word vectors to construct sentence vectors. Kedzie et al.^12^ averaged the word embeddings of sentences to get sentence embeddings. This approach assumes that each word has the same effect on the sentence, regardless of the particularity of the particular word. Based on the current and previous hidden states of the word embedment, Nallapati et al.^2^ applied RNN to compute the hidden state representation of each word position in turn, and then used the average, connected hidden states as sentence embedment. In contrast to the simple average word embedding representation, the approach of RNN considered the order of words.

We used the BERTSUM method to embedding sentence, utilized token embeddings, segment embeddings and position embeddings to take the information of the sentences into sentence embeddings.

## Method

The definition of the EDS task as follows. Given a single document $D$ that contains n sentences, $D = \{ s_{1} ,s_{2} , \ldots ,s_{n} \}$, where $s_{i} = \{ w_{i1} ,w_{i2} , \ldots ,w_{ij} \}$ is the $i\text{-th}$ sentence in the document and $w_{ij}$ is the $j - th$ word in the $i - th$ sentence. EDS can be seen as a sequence labeling task, which means that every sentence in the document is assigned a label $y \in \{ 0,1\}$ to suggest whether the sentence should be included in the summary. We introduce cluster algorithm into EDS task, which is used with sentence embeddings to reduce the high semantic redundancy sentences in summary. In this section, we will first introduce the BERTSUM^13^ model that is used for selecting sentences from document and present our cluster-EDS model.

### Score-BERT

**Figure 1:**
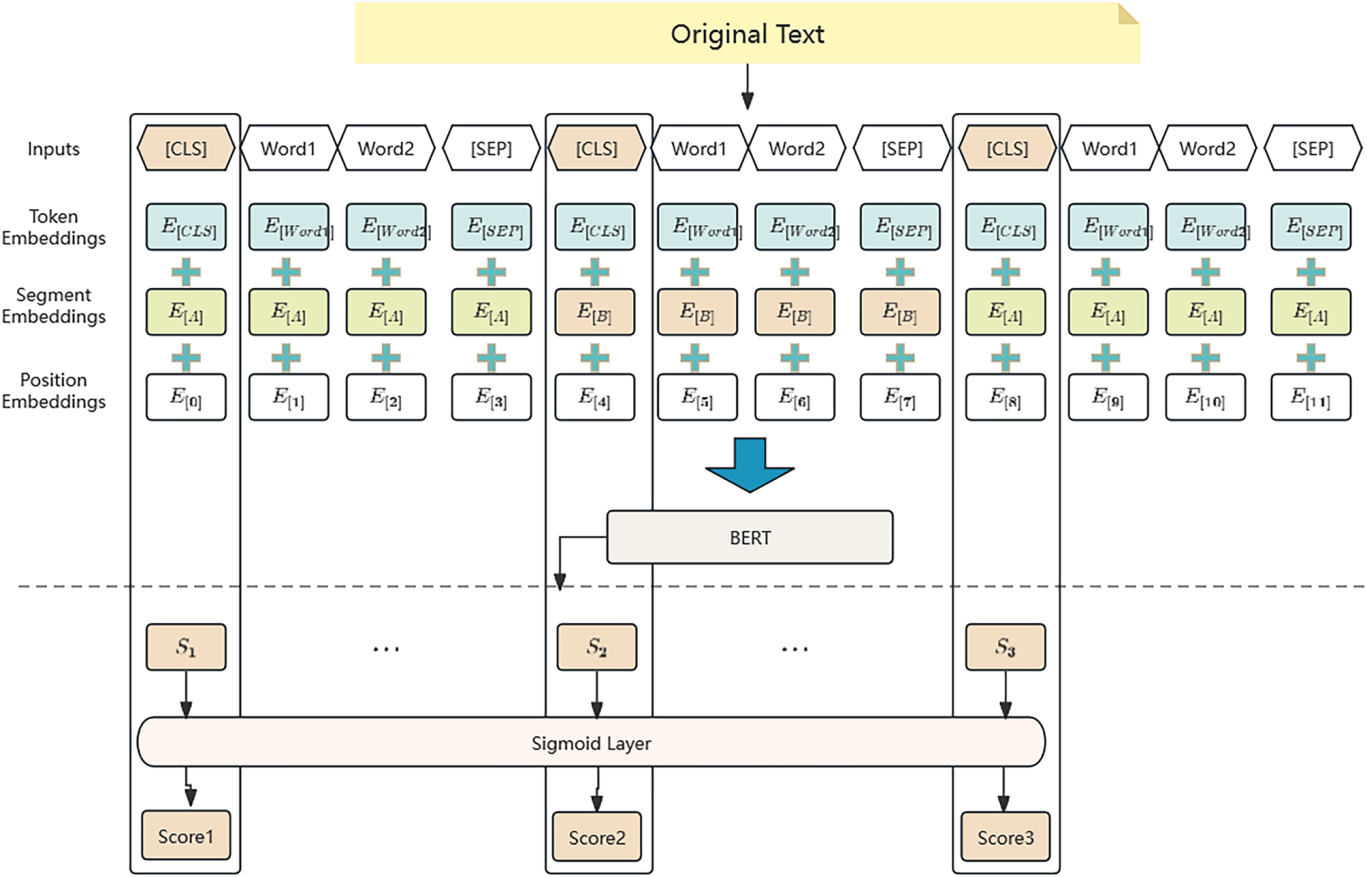
Score-BERT.

BERT model is used as the basis to build Score-BERT, which can score sentences. Score-BERT consists of two parts: encoder and sigmoid layer. In the encoder part, BERT model is used as the basis to encode the original text into semantic vector representation. Then the sigmoid layer takes a linear fully connected layer and uses sigmoid function to obtain a prediction score, which is used to select sentence. The Score-BERT is shown in Fig. 1.

### Cluster-EDS model

**Figure 2:**
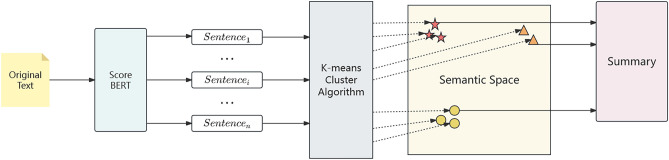
ClusterSum model.

Our model is based on Score-BERT. After Score-BERT selects n sentence vectors, the selected sentence vectors are mapped to the high-dimensional semantic space by K-means algorithm. K-means algorithm divides sentences into different clusters according to their semantics. Then, the sentences with higher sentence scores are selected from different clusters to form the summary, so that the selected sentences have less semantic redundancy, and the composed summary can have lower semantic redundancy. The model is shown in Fig. 2.

The K-means module receives the sentences embedding and the score from the Score-BERT, the K-means module then makes the sentences embedding map to high dimension semantic space and model will select the highest score of sentences from difference clusters, so the extracted summary is formulated by the sentences with high score and difference semantic.

### Text matching

Zhong et al.^4^ first applied method of text matching to extractive document summary task. Selected sentences formulated the candidate sets while passing through cluster algorithm. To be specific, candidate set is composed to sentences embeddings from different cluster.

We use the Siamese-BERT architecture to match the reference summary $D$ and the candidate summary $C$. Siamese-BERT consists of two BERTs with tied-weights and a cosine-similarity layer during the inference phase. We get the embeddings from reference summary $D$ and candidate summary $C$ by using the original BERT. We define $r_{D}$ and $r_{C}$ denote the embeddings of the reference summary $D$ and candidate summary $C$. Their similarity score is measured by $f(D,C) = \cos ({\text{r}}_{D} ,r_{C} )$.

## Experiment

### Datasets

We evaluate our approach on CNN/DailyMail dataset and get the better performance than baseline model. The CNN/DailyMail dataset is mainly derived from about 1 million news report data of CNN and the DailyMail. This dataset is a single-text abstract corpus, in which there are a large number of abstract chapters, and each chapter contains several abstract sentences, which are then briefly modified to form a corpus for text summarization technology. We follow the standard splits and preprocessing steps used in baseline, and resulting datasets contain 286,817 articles for training, 13,368 for validation, and 11,487 for test.

### Implementation detail and metrics

We use the BERTSUM to select sentences and sentence-encoding. Adam optimizer with warming-up is used and learning rate schedule follows Vaswani et al.^14^ as:

$$ lr = 2e^{ - 3} \cdot \min (step^{ - 0.5} ,stepwm^{ - 1.5} ) $$

where each step is a batch size of 8 and $wm$ denotes warmup steps of 10,000. We choose $r1 = 0$ and $r2 = 0.01$. When $r1 < 0.05$ and $0.005 < r2 < 0.05$, they have little effect on performance, otherwise they will cause performance degradation. We use the validation set to save three best checkpoints on the test set. Importantly, all experimental results listed in this paper are the average of three runs.

### Baselines and comparisons

To validate the effectiveness of the ClusterSum model. We choose some baseline models from two aspects to compare. On the one side, we choose the classic machine methods, on the other hand, we choose the-state-of-art deep learning model. Base on the above consideration, we compare our model with the following solid baselines for text summarization:

- LEAD-3: The method takes the first three sentences of the document as a summary.
- JECS: The method firstly selects sentences and then compresses each sentence by removing unnecessary words.
- LSTMPN: An extractive model based on LSTM and pointer network.
- LongformerExt: An extractive model based on Long Transformer.
- BERTSUMEXT: A method based on the pretrained model BERT.
- MatchSum: A model uses the approach of text matching to do the extractive summary task.

## Results

We test our model on the CNN/DailyMail. Rouge^15^ scores measure the summarization quality. The results of our Cluster-BERT model are presented in Table 1. As shown in Table 1, we list strong baselines with different learning methods.

**Table 1:** ROUGE scores measure the summarization quality.

Model	ROUGE-1	ROUGE-2	ROUGE-l
LEAD-3	40.34	17.70	36.57
JECS	41.70	18.50	37.90
LSTM + PN	41.85	18.93	38.13
Longformer-Ext	43.00	20.20	39.30
BERTSUMEXT	43.25	20.24	39.63
MatchSum	42.82	18.11	38.79
ClusterSum	44.22	20.26	40.12

ROUGE-1, ROUGE-2 and ROUE-l are used for reporting the unigram, bigram and longest common subsequence overlap with reference summaries.

The experimental results of our model based on the K-means algorithm model is shown in Table 1. The results show that the experimental performance on ROUGE-1, ROUGE-2, ROUGE-l outperforms all the baseline methods. Compare with MatchSum, the score of three metrics all have increased, which can conclude that the Cluster model is effective for extracting summary. These three metrics respectively evaluation the accuracy on unigrams, bigrams and longest common subsequence.

### Ablation study

We performed ablation experiments to discuss whether the K-means algorithm worked in ClusterSum model. Experiments are conducted on CNN/Daily Mail dataset. The models are presented as follow:

ClusterSum/K-means: ClusterSum model without K-means algorithm. In order to determine the influence of K-means on the model, we removed the K-means module. The specific approach is sorting the sentences scored by Score-BERT according to their scores, and selecting the three sentences with the highest scores as the summary.

Table 2 shows the performance difference caused by the K-means algorithm. We can see that our model well on the news datasets CNN/Daily Mail. Combining the advantages of K-means algorithm in reducing semantic redundancy of sentences, and experiment results, we can conclude that K-means algorithm can reduce the repeat sentences in semantic space to increase the quality of the extracted summary.

**Table 2:** The results of ablation study.

Model	ROUGE-1	ROUGE-2	ROUGE-l
ClusterSum/K-means	42.93	18.35	39.12
Cluster	44.22	20.26	40.12

## Conclusion

Focusing on the high semantic redundancy problem of extractive document summary task, we presented how cluster algorithm can be usefully in EDS task by reducing semantic redundancy sentences. We introduced a novel way to get the low semantic sentences to improve the performance of the EDS model. The experimental results show that the model we proposed get the higher score compare with baseline method in ROUGE score, so prove that cluster algorithm is effective to reduce the semantic redundancy.

In future work, we will continue to explore the approaches to reduce the semantic redundancy in EDS task as follows: different cluster algorithms how to influence the EDS model selecting sentences.

## Data Availability

The data used in the study are available from the corresponding authors upon request.
